# The effects of smoke-free legislation on acute myocardial infarction: a systematic review and meta-analysis

**DOI:** 10.1186/1471-2458-13-529

**Published:** 2013-05-31

**Authors:** Hualiang Lin, Hongchun Wang, Wei Wu, Lingling Lang, Qinzhou Wang, Linwei Tian

**Affiliations:** 1 Guangdong Provincial Institute of Public Health, Guangzhou, China; 2 Center for Disease Control and Prevention of Guangdong Province, Guangzhou, Guangdong, China; 3 Qilu Hospital of Shandong University, Jinan, China; 4 Department of neurology, Qilu Hospital of Shandong University, Jinan, China; 5 The Jockey Club School of Public Health and Primary Care, The Chinese University of Hong Kong, Hong Kong, China; 6 Shenzhen Municipal Key Laboratory for Health Risk Analysis, Shenzhen Research Institute of the Chinese University of Hong Kong, Shenzhen, China

**Keywords:** Smoke-free legislation, Acute myocardial infarction, Systematic review, Meta-analysis, Random effect

## Abstract

**Background:**

Comprehensive smoke-free legislation has been implemented in many countries. The current study quantitatively examined the reduction in risk of acute myocardial infarction (MI) occurrence following the legislations and the relationship with the corresponding smoking prevalence decrease.

**Methods:**

PubMed, EMBASE, and Google Scholar databases and bibliographies of relevant studies and reviews were searched for potential original studies published from January 1, 2004, through October 31, 2011. Meta-analysis was performed using a random effect model to estimate the overall effects of the smoking-free legislations. Meta-regression was used to investigate possible causes of heterogeneity in risk estimates.

**Results:**

A total of 18 eligible studies with 44 estimates of effect size were used in this study. Meta-analysis produced a pooled estimate of the relative risk of 0.87 (95% confidence interval (CI): 0.84 to 0.91). There was significant heterogeneity in the risk estimates (overall I^2^ = 96.03%, p<0.001). In meta-regression analysis, studies with greater smoking prevalence decrease produced larger relative risk (adjusted coefficient −0.027, 95% CI: -0.049 to −0.006, p=0.014).

**Conclusion:**

Smoke-free legislations in public and work places were associated with significant reduction in acute MI risk, which might be partly attributable to reduced smoking prevalence.

## Background

Tobacco smoking is projected to kill about one billion people worldwide in the 21st century [1]. The harmful effects of smoking are not only confined to active tobacco consumers, but also for those exposed to environmental tobacco smoke (ETS) [2], which is listed as the third leading cause of preventable poor health and premature deaths in the developed world [3]. According to the US Surgeon General’s report, tobacco smoking is a major population risk factor for coronary heart disease, the leading cause of deaths in the US [4]. Including acute myocardial infarction (MI), coronary heart disease has remained the second leading cause of deaths over the past three decades [4,5]. The harmful health effects of smoking has prompted many countries to enacted various smoking regulations in order to directly decrease exposure to environmental tobacco smoke and indirectly reduce active smoking, in hope to prevent and reduce smoking-related morbidity and mortality such as acute MI [6,7]. These smoking bans usually prohibited smoking activity in public and working places, such as restaurants, workplaces, and bars, although differnce existed among countries and cities [8]. A growing body of evidence has suggested that the rate of acute myocardial infarction significantly decreased after the introduction of the smoking ban regulations, usually within a short time period. However, the results published so far showed a large variation of the effect size, ranging from 5% to 70% [9]. On the other hand, the relationship of this reduction with the corresponding smoking prevalence change remained unknown. This study performed a systematic literature review and meta-analysis with the aim to estimate the overall effect size of smoke-free legislations on the risk of acute MI in the general population, and to investigate the relationship between reduction in smoking prevalence and the acute MI rate change after the smoking-free legislations.

## Methods

The literature search was conducted to find potential studies published from January 1, 2004, through October 31, 2011. We used the MEDLINE, EMBASE, and Google Scholar database without restrictions and we included articles that were ahead of publications. The following keywords were used in the literature searching: “smoking ban” and “heart” or ”myocardial infarct”. Moreover, we searched for the keywords in headers and abstracts and also performed a manual search of references cited in the selected articles and published reviews to look for any additional relevant studies. A total of 19 studies were identified, of which, 18 had been published in peer-reviewed journals and 1 had not. The latter study did not provide enough information for us to calculate relative risk and confidence interval, was thus not included in the meta-analysis.

Two individual studies included the city of Pueblo, Colorado: the first was on the effects for the first 18 months after the law being implemented [10] and the second was after 36 months [11]. And two studies reported that of the City of Graubuenden, Switzerland. One was one year after the smoking ban and the other one was two years after the regulation [12,13]. Data of Piedmont, Italy were used as part of one study, which was for the effect of 2 months after the law went into effect [14] and another study examined the effect of the smoking ban in Piedmont after 6 months of the implementation [15]. These studies were initially treated as independent observations in this study, although there was some overlaps in the baseline information. And in the sensitivity analysis, we only included the more recent studies for these cities in order to check the robustness of the result estimation. Information of New York was also included in two studies: one including the residents aged 35 years and over [16], the second including those of 45 years and above [17], only the former one was included in this analysis as it included the information of the latter one. One study [14] reported results from four Italian regions, one of which had already been reported individually [15]; the results for the other three regions were used separately into the meta-analysis.

Some studies reported separate relative risks for different age subgroups and sexes, these estimates were entered separately into the meta-analysis. The meta-analysis was therefore based on a total of 44 estimates of relative risk obtained from 18 individual studies.

When population data were not provided in the studies, they were obtained from the relevant country's census records. If the smoking prevalence information was not available in the study, the information was extracted from various sources. For example, the studies in the USA, data were obtained from a report by American Lung Association [18]. And information of Toronto, Ontario Canada was obtained from [19], England from [20], Italy from [21], Christchurch, New Zealand from [22], Graubuenden, Switzerland from [23,24].

All analyses were performed using R software. As the studies were conducted in different countries and circumstances, we utilized a random-effect meta-analysis which allowed for non-random variability in effect estimates between the studies. A forest plot was produced to illustrate the contributions of each individual study in terms of their estimated effect size and the 95% confidence interval (CI). To examine potential publication bias, we plotted the standard errors (SEs) of the studies against the corresponding effect sizes. We assessed the possibility of publication bias by visually measuring the asymmetry of the funnel plot. A funnel plot could allow for widening 95% CI lines with decreasing study size. If there was no significant bias, 95% of the studies would lie within these lines and, in the absence of small study bias, the plotted results should be symmetrical. We also used the Egger's linear regression method to statistically examine the symmetry. Meta-regression analysis was further conducted to examine whether the estimate of relative risk (RR) was associated with such factors as population size, study location (U.S. or non-U.S.), publication year and smoking prevalence decrease.

## Results

This systematic review identified 18 eligible studies (9 conducted in US, 3 in Italy, 2 in Canada, 2 in Switzerland, 1 in Great Britain and 1 in New Zealand). All of the studies were based on acute MI hospitalization except that three studies used acute MI mortality data [17,25,26].

All of the studies included a pre- and post- comparison of the acute MI hospitalization or mortality rate. Eleven also included a geographical comparison area where legislation had not yet been implemented [3,7,11,13,17,25-29]. These studies were all ecologic in the study design, and they varied widely in population size (ranging from 9,100 to more than 15 million), and in the duration of post-ban observation (from 2 to 36 months, with only 3 studies reporting results of within 12 months [14,15,28]).

**Figure 1:**
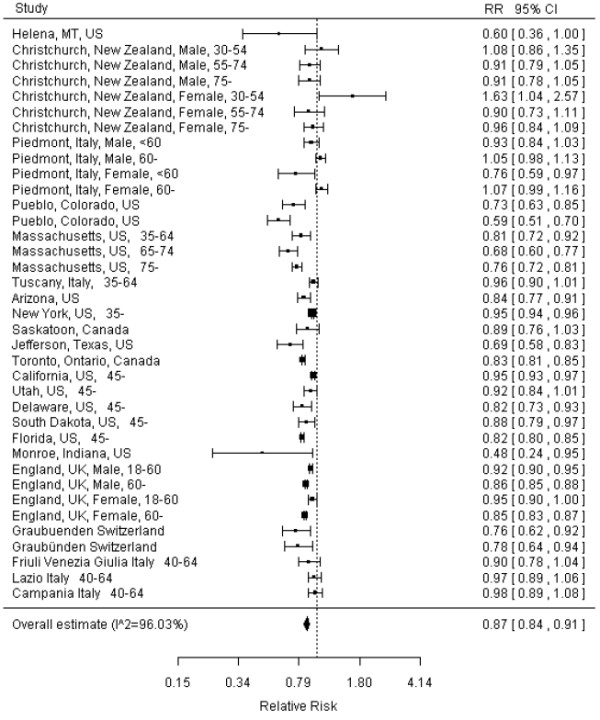
Forest plot of random effects meta-analysis of studies examining the effect of smoke-free legislation on acute myocardial infarction.

As shown in Figure 1, the overall relative risk of acute MI before and after smoking-free legislations was 0.87 (95% CI: 0.84-0.91), suggesting that the smoking-free legislations might have reduced acute MI occurrence by 13% on average. The study in New York observed an 8% reduction in hospitalization for acute myocardial infarction [16]. The authors estimated that the decline would have been 19% if there had been no prior local smoking restrictions. When we re-ran the analysis inputting a 19% reduction rather than 8%, the pooled estimate remained as 87% (95% CI: 83% to 90%).

**Figure 2:**
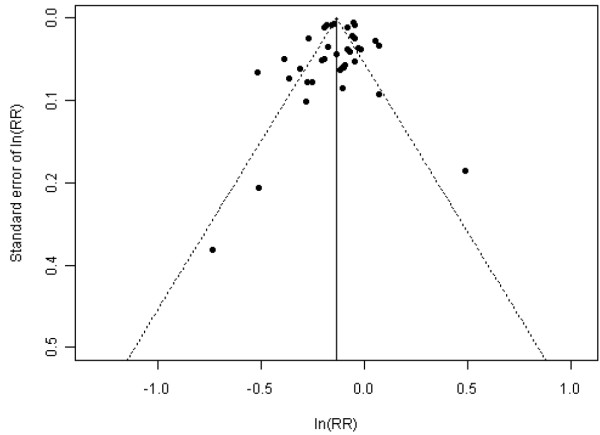
Funnel plot to illustrate possible small study bias among studies examining the effect of smoke-free legislation on acute myocardial infarction.

The overall I^2^ was 96.03%, suggesting significant heterogeneity in the risk estimate in different areas (p<0.001). The publication bias of the studies was examined by analyzing the funnel plot and Egger's regression test. As shown in Figure 2, the funnel plot appeared asymmetrical, and Egger's regression test suggested significant asymmetry (bias coefficient=4.05, p=0.0003), indicating either publication bias or heterogeneity that cannot be simply explained by a random-effect meta-analysis.

In the sensitivity analysis, we excluded the studies with acute MI mortality as the outcome, the result estimate did not change appreciably (RR=0.89, 95% CI: 0.85-0.93). When we included only the more recent studies for the same city in the meta-analysis, the effect remained similar (RR=0.84, 95% CI: 0.84-0.92).

**Figure 3:**
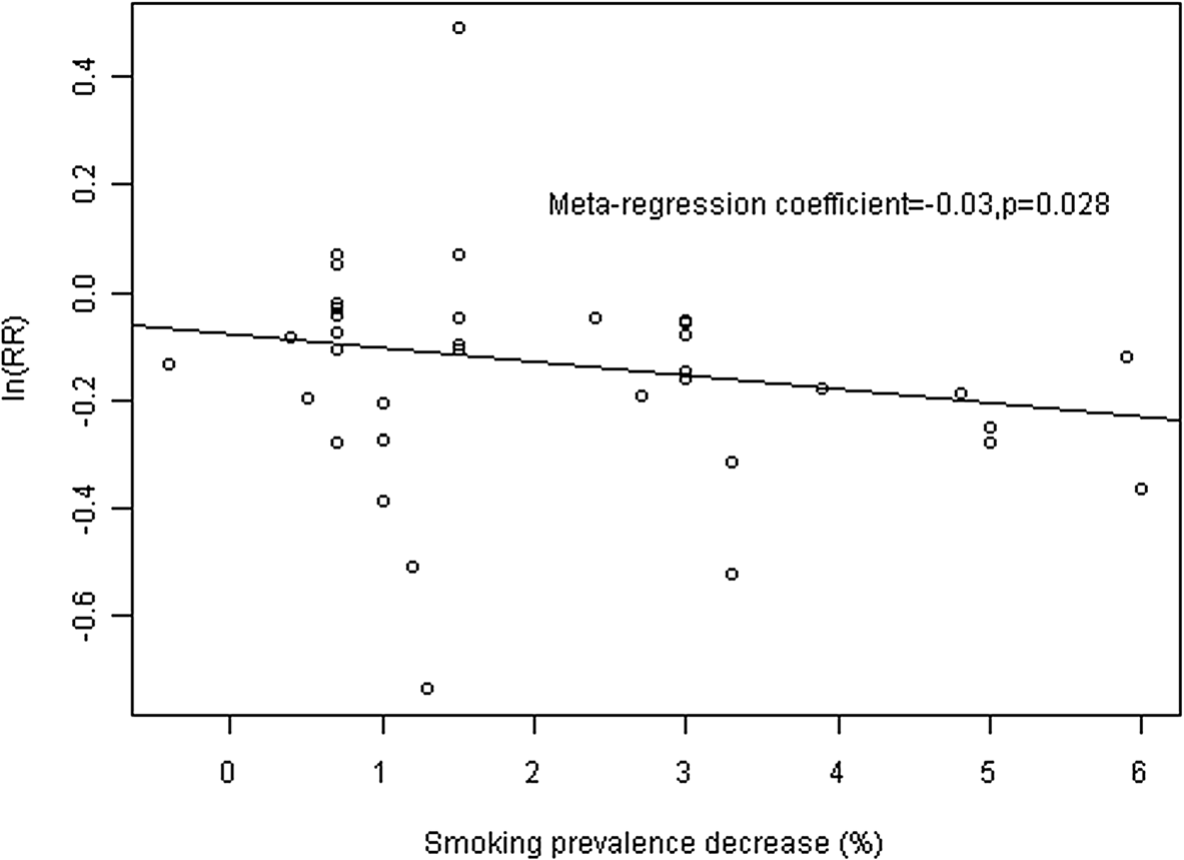
Relationship of AMI decrease with smoking prevalence rate decrease.

Further meta-regression analysis showed that the heterogeneity was partially explained by the study location and smoking prevalence rate change. In the univariate meta-regression analysis, study location (US vs. non-US, p=0.001) and smoking prevalence rate decrease (p=0.028) were significantly associated with estimated effect size (Table 1), and they remained significant predictors after adjustment for other study covariates in the multivariate analysis. There was a dose gradient that the higher the smoking prevalence decreases following the implementation of the smoking-free legislations, the greater the reduction in acute myocardial infarction occurrence (Figure 3).

**Table 1:** Random effects meta-regression analysis of studies examining the effect of smoke-free legislation on acute myocardial infarction

**Variable**	**Univariate model**		**Multivariate model**	
	**Coefficient (95% CI)**	**p value**	**Coefficient (95% CI)**	**p value**
Study population				
≥500 000				
<500 000	0.060(−0.023 to 0.142)	0.157	0.048(−0.022 to 0.118)	0.176
Study location				
US				
Non-US	0.133(0.057 to 0.209)	0.001	0.124(0.049 to 0.199)	0.001
Publication Year	−0.018(−0.042 to 0.006)	0.134	−0.005(−0.028 to 0.018)	0.683
Smoking decrease	−0.028(−0.052 to-0.003)	0.028	−0.027(−0.049 to −0.006)	0.014

## Discussion

Since 2004, when the first report of a significant drop in acute MI rate related to the smoking ban in public and work places was published for the town of Helena, Montana [28], there has been an increasing number of demonstrations of this topic in various countries. The current study provided an up to date meta-analysis of the existing literatures on the effectiveness of smoking-free legislation on the risk of acute MI. Similar to the pooled estimates published in previous meta-analyses [30,31], this study of 18 studies (44 estimates) indicated a significant reduction of acute MI rate after the implementation of smoking bans in public and work places. To our knowledge, our study was the first to examine the association between the acute MI reduction and the corresponding smoking prevalence decrease, which provided further evidence of the association of environmental tobacco smoke with acute MI.

The findings from this meta-analysis indicated that the smoking-free legislations were associated with a reduced risk of acute MI in general population. Overall, the risk of acute MI decreased by 13%, a moderate effect size compared with previous estimates of a 27% decrease by Dinno [32], an updated 19% reduction by Glantz [30] and 17% by two other studies [31,33]. However, our estimate was consistent with a 15% drop by one study [33]. And this was supported by Pechacek and Babb, which estimated 18% to 19% as the maximum impact that could be expected to be associated with smoking ban [34]. Some studies reported separate estimates for different subpopulations, only those with significant reductions were included by previous meta-analyses, which might be one reason for the relatively higher estimate by previous meta-analyses.

We noticed that the results published so far showed a large variation: studies with smaller population in the United States [10,11,28,35] usually reported larger reductions ranging from 27% to 40%, while larger studies usually reported relative modest reductions: 5% in New Zealand [36], 8% in New York [16], 13% in four Italian regions [14], and 13% in Switzerland [12]. At the same time, some subgroup estimates did not find significant reduction (for example, among people aged 60 years old and above in Piemont, Italy [15], population aged 30–54 years old in Christchurch, New Zealand [36]). The discrepancy among these studies could in part be attributable to different compliance with the legislation and different changes in smoking behavior among different countries.

Our meta-regression analysis showed that the reduction in acute MI risk was greater in studies with higher smoking prevalence rate decreases, suggesting that the protective effect of legislation could be directly attributed to the corresponding smoking rate decrease. This was corroborated by the study in Arizona, US, which demonstrated a 3.9% decrease in smoking prevalence, inducing a 13% reduction of acute MI hospitalization [7].

There were several possible pathways linking the smoking ban regulations to acute MI reduction. The smoking-free policy could reduce the amount of cigarette consumption among the active smokers, encourage smoking quitting, enhance the awareness of the public about the harmfulness of smoking, and more importantly reduce environmental tobacco smoke exposure among the passive smokers. Thus, the beneficial effects could be expected to be in a long-run fashion, besides the short-term effects [37].

The implementation of smoking ban law had been reported to result in significant decline in environmental tobacco smoke [16,38]. For example, in New York, after the implementation of the statewide smoking restriction law, population exposure to environmental tobacco smoke declined by nearly 50% and cotinine levels in the saliva from New York State adults declined from 0.078 ng/ml to 0.041 ng/ml [11,16]. In UK and Ireland, a 95% reduction in ETS among the general population was reported 9 months after the smoking ban [39]. In Scotland, the indoor fine particulate matter (PM_2.5_) concentration declined by 86% two weeks after the Scottish legislation [38]. In Ireland, salivary cotinine concentration among hotel workers fallen by 69% and air nicotine concentration dropped by 83% [40].

This finding strengthened our knowledge that passive smoking was a serious risk factor for acute myocardial infarction and that its elimination reduced the acute MI occurrence [41,42]. One percentage decrease in smoking prevalence was estimated to reduce 2.8 percentage of acute MI rate on average. And this association was biologically possible. Exposure to ETS could rapidly induce platelet aggregation, thrombosis, endothelial dysfunction and inflammation, and these effects were comparable to those suffered by active smokers [41,42]. These effects were estimated to increase the risk of acute myocardial infarction by around 10% globally [43], which was in line with the current estimate. As this risk factor was relatively easily modifiable, the expansion of the smoke-ban policy in public and working places were expected to have a pivotal public health significance.

The strength of the present study included the up to date pooled estimation of the relationship of acute MI risk with the smoke-free legislations and the examination of the correlation of this reduction with the corresponding smoking rate decreases. Meanwhile, a few limitations of this study should be noted. All the included studies were the ecological studies in study design nature, which caused uncertainty in causal inference. Nonetheless, when taken in aggregate, these studies offered evidence that smoking bans were followed by significant reduction in the rate of acute MI in the general population. A recent study reported contradicting results on the impact of smoking ban on acute MI in 387 US counties when adjusting for non-linear secular trend in acute MI compared with the model adjusting for linear secular trend [8]. However, most of the studies included in this meta-analysis did not consider non-linear trend of acute MI rate [44], which might have caused concern in our estimate. Future studies should consider the non-linear secular trend in acute MI occurrence. Temporal trend of acute MI rate might be influenced by factors other than smoking exposure, such as long-term trend, other air pollutants, atmospheric temperature, influenza epidemics, changes in diagnostic standards and preventive strategies, which were not taken into account in this meta-analysis. It was also possible that the reduction in acute MI risk could be due to the reduced amount of cigarettes smoked. It should also be noted that the smoking rate used in the analysis came from diverse sources, where the definition of smoking might be different, however, we used the difference of smoking prevalence before and after the smoking free legislation as the indicator, so this should not have influenced the result seriously.

## Conclusion

The present meta-analysis of 18 individual studies with 44 estimates suggests that smoking bans are associated with significant reduction in AMI occurrence, and that this reduction might be partly due to the declines in smoking prevalence following the smoke-free legislations. Health policy makers, health care professionals should strongly advocate more comprehensive smoking restrictions in public areas and workplaces.

### Statement

The study adheres to the PRISMA guidelines for systematic reviews.

## Competing interests

The authors declare that they have no competing interests.

## Authors’ contributions

HLL, QZW and LWT conceived and designed the study; HLL, HCW, LLL and WW performed the analysis and drafted the manuscript. All authors approved the submitted version of the manuscript.

## Pre-publication history

The pre-publication history for this paper can be accessed here:

http://www.biomedcentral.com/1471-2458/13/529/prepub
